# Maintenance therapy in NSCLC: why? To whom? Which agent?

**DOI:** 10.1186/1756-9966-30-50

**Published:** 2011-05-06

**Authors:** Silvia Novello, Michele Milella, Marcello Tiseo, Giuseppe Banna, Diego Cortinovis, Massimo Di Maio, Marina Garassino, Paolo Maione, Olga Martelli, Tiziana Vavalà, Emilio Bria

**Affiliations:** 1 Thoracic Oncology Unit, University of Turin, AOU, San Luigi Orbassano, Italy; 2 Department of Medical Oncology, Regina Elena National Cancer Institute, Rome, Italy; 3 Department of Medical Oncology, AOU Parma, Italy; 4 Department of Medical Oncology, Cannizzaro Hospital Catania, Italy; 5 Department of Medical Oncology, San Gerardo Hospital Monza, Italy; 6 Clinical Trials Unit, National Cancer Institute Naples, Italy; 7 Department of Medical Oncology, Fatebenefratelli and Oftalmico Hospital, Milan, Italy; 8 Department of Medical Oncology, San Giuseppe Moscati Hospital Avellino, Italy; 9 Department of Medical Oncology, San Giovanni-Addolorata Hospital Rome, Italy

## Abstract

Maintenance therapy is emerging as a treatment strategy in the management of advanced non small cell lung cancer (NSCLC). Initial trials addressing the question of duration of combination chemotherapy failed to show any overall survival benefit for the prolonged administration over a fixed number of cycles with an increased risk for cumulative toxicity. Nowadays several agents with different ways of administration and a different pattern of toxicity have been formally investigated in the maintenance setting. Maintenance strategies include continuing with an agent already present in the induction regimen or switching to a different one. Taking into consideration that no comparative trials of maintenance with different chemotherapy drugs or targeted agents have been conducted, the choice and the duration of maintenance agents is largely empirical. Furthermore, it is still unknown and it remains an open question if this approach needs to be proposed to every patient in the case of partial/complete response or stable disease after the induction therapy. Here, we critically review available data on maintenance treatment, discussing the possibility to *tailor *the right treatment to the right patient, in an attempt to optimize costs and benefits of an ever-growing panel of different treatment options.

## Introduction

Lung cancer is the leading cause of cancer mortality in USA and worldwide more than one million people die from this disease every year: the overall 5-year relative survival rate measured by the Surveillance Epidemiology and End Results program in USA is 15.8% [1]. Approximately 87% of lung cancer cases are Non Small Cell Lung Cancer (NSCLC) and the majority of patients presents with advanced stage disease at diagnosis [2,3]. In two independent phase III trials the addition of bevacizumab to standard first-line therapy was shown to improve both overall response rate (ORR) and PFS, although OS advantage was demonstrated in only one of these studies [4,5]. In combination with platinum-based chemotherapy, cetuximab has also demonstrated a small statistically significant OS advantage as compared to chemotherapy alone [6]. Second-line treatment has been shown to improve survival and to palliate symptoms: approved treatment options include cytotoxic chemotherapy (docetaxel or pemetrexed) or epidermal growth factor - EGFR tyrosine kinase inhibitors (erlotinib or gefitinib) [7,8]. However, only approximately 50% of the patients will be able to receive second-line therapy, mainly because of the worsening of clinical conditions [9].

One of the strategies, that has been extensively investigated in recent years in order to improve current clinical results in advanced NSCLC, is the maintenance therapy. Here, we review available data on maintenance treatment, discussing about the possibility to *tailor *the right treatment to the right patient, in an attempt to optimize costs and benefits of an ever-growing panel of different treatment options.

## Maintenance therapy: working definitions

The U.S. National Cancer Institute's medical dictionary defines maintenance therapy as "any treatment that is given to keep cancer from progressing after it has been successfully controlled by the appropriate front-line therapy; it may include treatment with drugs, vaccines or antibodies, and it should be given for a long time". Maintenance therapy has also been referred to as "consolidation therapy" or "early second-line therapy", depending on treatment type and timing of the specific therapeutic agent employed [10]. The latter definition is probably the least appropriate, because "second-line" implies a disease progression event, which, by definition, is not the case for the maintenance setting and the term "switch maintenance" (used in the National Comprehensive Cancer Network - NCCN - Clinical Practice Guidelines) appears more precise[11].

Currently, for advanced NSCLC the options to continue treatment after first-line induction include: 1) continuing induction therapy for a fixed number of additional cycles over the standard or, when possible, until progression; 2) continuing only the third-generation non-platinum compound used in the induction regimen; 3) switching to a different agent after induction therapy.

## Continuing first-line induction therapy

The first American Cancer Society of Clinical Oncology (ASCO) guidelines, published in 1997, addressed the appropriate duration of therapy in advanced NSCLC recommending no more than eight cycles, even if in most clinical trials the median number of delivered cycles is typically three or four [12]. Four trials clarified that were no response, survival or QoL differences between short versus longer treatments in advanced NSCLC but an increased risk for cumulative toxicity only (Table 1) [13-16]. As consequence ASCO changed recommendations regarding the appropriate duration of therapy in 2003, stating that treatment should have been stopped at four cycles for non responders patients and no more than six cycles should have been administered for any patient; no major changes for this specific issue were reported in the ASCO guideline update in 2009 [17,18].

**Table 1:** Randomized or prolonged therapy in older chemotherapy regimens

Trial	N	Treatment arm	Completed treatment*	PFS	p	OS	P	References
**Smith 2001**	308	3 vs 6 mytomicin/cisplatin/vinblastine	72% vs 31%	5 mo vs 5	0.4	6 mo vs 7	0.2	[13]

**Socinski 2002**	230	4 Carboplatin/Paclitaxel vs Carboplatin/Paclitaxel until PD	57% vs 42%receiving >4cycles#	-	-	6.6 mo vs 8.5	0.63	[14]

**Von Plessen 2006**	297	3 vs 6 Carboplatin/Vinorelbine	78% vs 54%	16 wks vs 21	0.21	28 w vs 32	0.75	[15]

**Park 2007**	314	4 vs 6 cycles platinum-based therapy	68% vs 92%	4.6 mo vs 6.2	0.001	14.9 mo vs 15.9	0.41	[16]

PFS: progression free survival, OS: overall survival; PD: progressive disease; mo: months; wks: weeks;

*Percentage of patients who received the all planned courses of therapy

#the percentage of grade 2-4 neuropathy in four arm cycles was 19% versus 43% in eight arm cycles.

## Continuing the same non-platinum compound used in the induction regimen

In patients responding or stable after the induction, a maintenance strategy should be to continue the same therapy withholding platinum, in an attempt at consolidating disease control and increasing survival, maintaining tolerability within acceptable limits.

The European Cooperative Oncology Group conducted a phase III trial testing gemcitabine maintenance versus best supportive care (BSC) in 350 patients with complete/partial response or stable disease after four cycles of gemcitabine/cisplatin induction, randomized in a 2:1 ratio. Sixty one percent of patients (among 73% of responders after the induction) were randomized: during the maintenance period, patients received a median of three cycles of gemcitabine (range: 0-38 cycles). Median TTP was significantly longer in the gemcitabine arm both throughout the study (6.6 versus 5 months, p < 0.001) and during the maintenance period (3.6 versus 2 months, p < 0.001). Median OS in the gemcitabine arm was 13 months, compared to 11 months in the BSC arm (p = 0.195). In terms of toxicity, the most important difference between the two arms during the maintenance phase was the need for red blood cells transfusions (20% in the gemcitabine arm versus 6.3% in the BSC arm, p = 0.018) [19]. Another phase III trial comparing gemcitabine versus BSC as maintenance therapy for patients not progressing after 4 cycles of gemcitabine/carboplatin induction was recently presented. Two hundred and fifty five patients (among 519 enrolled) were randomized; median PFS was 3.9 months (95% CI: 3.3-5.6) for the experimental arm and 3.8 months (95% CI: 2.6-5.5) for the BSC arm; median OS (primary end point) was 8 months (95% CI: 6.0-10.2) for the gemcitabine maintenance arm and 9.3 months (95% CI: 7.7-12.7) for the BSC arm, without any statistical difference [20]. In a third trial employing gemcitabine or erlotinib maintenance after 4 cycles of gemcitabine/cisplatin induction and with a preplanned II-line treatment option (pemetrexed), PFS (primary end point) by independent review was significantly prolonged by both G (HR 0.51, 95% CI 0.39-0.66) and E (HR 0.83, 95% CI 0.73-0.94), as compared to O. OS data are not yet mature [21]. Belani et al. treated 401 patients with carboplatin and paclitaxel for 16 weeks; responding patients were then randomly assigned to receive weekly paclitaxel maintenance or BSC. Response was seen in 130/390 evaluable patients, who were deemed eligible for randomization into the maintenance phase, during which only 23% completed four cycles. Median TTP (primary endpoint) was 38 weeks in the paclitaxel arm versus 29 weeks in the BSC arm (p not reported); median OS was 75 and 60 weeks in the paclitaxel and BSC arm, with 1-year survival rates of 72% and 60%, respectively. During maintenance therapy, 86% of patients in the chemotherapy arm experienced at least one adverse event and 45% reported at least one grade 3 or 4 adverse event [22].

## Switching to a different agent after a platinum-based induction

According to the Goldie-Coldman hypothesis, showing that even the smallest detectable cancers contain at least one drug resistant clone and that increasing numbers of resistant clones emerge as tumors grow and progress, a rational strategy would be to use all effective drugs as early as possible in the treatment program [23,24]. Different non-cross-resistant agents have been used as a maintenance strategy after a defined number of induction cycles with a platinum-based regimen in several randomized clinical trials (Table 2).

**Table 2:** Studies with switch to a different agent after a platinum-based induction

First Author (N of randomized pts to maintenance)	Maintenance Schema	Primary End Point	Median PFS (mo)	P value	Median OS (months)	P value	References
**Fidias P**. (309)	Immediate vs delayed docetaxel	OS	5.7 vs 2.7	0.0001	12.3 vs 9.7	0.08	[26]

**Ciuleanu T**. (663)	Pemetrexed vs placebo	PFS	4.3 vs 2.6	0.0001	13.4 vs 10.6	0.012	[27]

**Cappuzzo F**. (889)	Erlotinib vs placebo	PFS	12.3 vs 11.1	0.0001	12 vs 11	0.063	[31]

**Perol M**. (464)	Gemcitabine vs erlotinib vs placebo	PFS	3.7 vs 2.8 vs 2.1	nr	HR 0.86 vs 0.81	na	[21]

Kabbinavar F.* (768)	Bevacizumab ± Erlotinib	PFS	4.8 vs 3.7	0.006	Na	na	[32]

**Gaafar RM **(173)	Gefitinib vs placebo	OS	4.1 vs 2.9	0.0015	Na	na	[33]

*In this trial bevacizumab was already present in the induction therapy

nr: not reported, na: not available

### Vinorelbine versus placebo

Westeel et al. designed a trial testing vinorelbine maintenance in stage IIIB and IV NSCLC after induction with mitomycin, ifosfamide and cisplatin (MIC). Nearly 600 patients were recruited and 181 were randomized to receive vinorelbine maintenance or BSC for up to 6 months. Mean duration of therapy was 13.8 months and 23% of patients completed 6 months of vinorelbine: in the majority of cases treatment interruption was due to disease progression (38%) or treatment toxicity (21%). The HR for OS, after adjusting for stage, was 1.08 (95% CI = 0.79 to 1.47; p = .65) and median OS was 12.3 months in both arms. One- and 2-year survival rates were 42.2% and 20.1% in the vinorelbine arm and 50.6% and 20.2% in the BSC arm respectively (log-rank P = .48). No difference in PFS was observed (HR = 0.77, 95% CI = 0.56 to 1.07; p = .11; median PFS 5 months with vinorelbine and 3 months in the BSC arm) [25].

### Immediate versus delayed docetaxel

Fidias and coll. conducted a phase III trial randomly assigning patients with objective response or stable disease after four cycles of gemcitabine/carboplatin first-line chemotherapy to immediate ('maintenance') docetaxel or a "delayed" second-line docetaxel, initiated at the time of disease progression. A total of 566 patients were enrolled and 309 patients with non-progressive disease were randomized. Among 153 patients assigned to immediate docetaxel, 145 (94.8%) received at least one treatment cycle and among 154 patients assigned to the to "delayed docetaxel", 98 (62.8%) patients initiated therapy. Reasons for not initiating the planned second-line included toxicity from previous treatment, decline in PS, and investigator's decision. The median number of docetaxel cycles administered in both arms was 4.4. There was a statistically significant advantage in PFS (5.7 versus 2.7 months, p = .0001) with maintenance docetaxel but, despite a 3-months improvement in median OS (primary endpoint), the difference did not reach statistical significance (12.3 vs. 9.7 months, p = .0853)[26].

### Pemetrexed versus placebo

Patients with advanced NSCLC with a disease control after four cycles of platinum-based therapy (not including pemetrexed) were randomized (2:1) to pemetrexed maintenance or placebo, until disease progression. A total of 663 patients were randomized and, among patients randomized to pemetrexed, 48% received more than 6 cycles of chemotherapy and 23% received more than 10 cycles. In the intent-to treat patient population, pemetrexed significantly improved both PFS (primary end point; HR = 0.50, 95% CI: 0.42 to 0.61, p < 0.0001; median PFS 4.3 and 2.6 months, respectively) and OS (secondary end point; HR: 0.79, 95% CI: 0.65 to 0.5, p = 0.012; median OS 13.4 and 10.6 months, respectively) as compared with placebo [27]. A pre-specified analysis by histology was incorporated into the protocol showing consistent data with other recent studies using pemetrexed [28,29]. In the non-squamous subgroup, pemetrexed strikingly improved PFS (HR = 0.44, 95% CI:0.36 to 0.55 median PFS 4.5 and 2.6 months, respectively) and OS (HR 0.70 95% CI: 0.56 to 0.88; p = 0.02, interaction p value 0.033) with a median survival advantage of 5 months (15.5 months versus 10.3 months). A significant delay in symptom worsening was observed on the pemetrexed arm especially for pain and hemoptysis.

### Erlotinib versus placebo

Cappuzzo et al. evaluated the benefit of the EGFR tyrosine kinase inhibitor erlotinib as maintenance therapy in a phase III trial comparing erlotinib versus placebo, in patients who had not experienced disease progression after four cycles of platinum-based therapy. The primary endpoints were PFS in the overall population and PFS in patients whose tumors had EGFR protein overexpression (as determined by immunoistochemistry - IHC). Patients assigned to erlotinib experienced a statistically significant improvement in PFS in both the intent-to treat (HR = 0.71 95% CI: 0.62 to 0.82 p < 0.0001; median 12.3 versus 11.1 weeks, respectively) and the EGFR IHC positive patient populations (HR = 0.69, 95% CI: 0.58 to 0.82; p < 0.0001). In the ITT population, patients assigned to the erlotinib arm experienced a statistically significant improvement in OS (HR = 0.81, 95% CI:0,70 to 0,95; p = 0.0088; median OS 12.0 versus 11.0 months, respectively). OS benefit was consistent across all patient subgroups; however, OS data for the EGFR mutation-positive population are highly censored and there was extensive crossover of EGFR-mutated patients assigned to placebo to EGFR TKIs in second-line therapy (16 of 24 patients, 67%). Patients who had stable disease after first-line chemotherapy seemed to have a more pronounced OS benefit with maintenance erlotinib (median 11.9 versus 9.6 months, respectively; HR 0.72, 0.59-0.89; p = 0.0019) than those who had complete or partial response to induction treatment (median 12.5 versus 12.0 months, respectively; HR 0.94,0.74-1.20; p = 0.618)[30,31].

### Gemcitabine or erlotinib versus placebo

Perol et al. recently presented the results of a phase III trial comparing maintenance gemcitabine or erlotinib versus placebo in patients, whose tumors had not progressed following platinum-based chemotherapy. Among 834 patients who received induction chemotherapy, 464 were randomized to observation (O, N = 152), erlotinib (E, N = 153) or gemcitabine (G, N = 149). A predefined second-line therapy (pemetrexed) was built-in in the study design in all arms. PFS (primary end point) by independent review was significantly prolonged by both G (HR 0.51, 95% CI 0.39-0.66) and E (HR 0.83, 95% CI 0.73-0.94), as compared to O. OS data are not yet mature [21].

### Bevacizumab/erlotinib versus bevacizumab

The ATLAS study is a phase III study designed to build on the use of bevacizumab as maintenance therapy for patients treated with an induction containing the same monoclonal antibody together with a platinum-based treatment. Specifically, the ATLAS study sought to determine whether the addition of erlotinib to bevacizumab could be more effective than bevacizumab alone, when used in the maintenance setting. A total of 1,160 patients were enrolled and, after completion of four induction cycles, non-progressing patients (N = 768, 66%) were randomized to receive bevacizumab alone or in combination with erlotinib. This trial was stopped after a planned interim efficacy analysis, reaching an improvement in PFS, that was the primary end point. Patients receiving erlotinib and bevacizumab experienced a superior PFS compared to bevacizumab alone (HR = 0,71, 95% CI: 0.58 to 0.86, p = 0.006; median PFS 4.8 and 3.7 months, respectively). Post-study therapy was at discretion of the investigator, and the rates of subsequent therapies on the erlotinib/bevacizumab and bevacizumab arms were 50.3% and 55.5%, respectively. In both arms 39.7% of patients received erlotinib as subsequent therapy. At the time of primary analysis of PFS 31% of patients had events and no further analyses of OS are planned, due to loss of patients to follow up [32].

### Gefitinib versus placebo

The European Organization for the Research and Treatment of Cancer 08021 evaluated the role of Gefitinib (G) administered after standard first-line chemotherapy in patients with advanced NSCLC. Initially all stable and responding patients were eligible for the study, which was then amended to require also evidence of EGFR protein expression by IHC. This resulted in recruitment slowing down, which ultimately led to premature study closure, after inclusion of 173 patients. The results showed a statistically significant difference in PFS (primary end point; 4.1 and 2.9 months, HR = 0.61, [95% CI 0.45,0.83], p = 0.0015) favouring G. The continuous administration of G following platinum-based chemotherapy in patients with advanced NSCLC was well tolerated. Based on 149 of the required 514 deaths, no difference in OS could be detected [33].

## 'Tailoring' maintenance therapy: which agent to which patient and future perspectives

As highlighted in the previous paragraphs, evidence on the continued (maintenance) use of the same third-generation agent employed in the induction regimen remains inconclusive with respect to gemcitabine and frankly negative in terms of cost/benefit ratio with respect to weekly paclitaxel [20-22,34]. Nowadays, available data about pemetrexed in maintenance setting do not answer to the question if this approach could be useful in those patients responding to a first line with platinum compound and pemetrexed and the answer will be available soon from a randomized trial comparing pemetrexed versus placebo in patients who do not progress following four cycles of pemetrexed plus cisplatin [35]. Positive data in terms of cost-effectiveness switching to pemetrexed, which employment in non-squamous NSCLC is really cost-effective, are driven by its impact on PFS and OS [36]. This is indeed a crucial point: resources use and costs involved with this new paradigm in the clinic, would all argue for a meaningful improvement in survival as a critical necessity from a practical standpoint. As a consequence, the usefulness of maintenance therapy has to be based on a clearly defined, reproducible and measurable endpoint. Using PFS as the basis for the adoption of a new therapeutic approach, may be considered as a limitation due to the variability in the definition of progression and frequency of response assessment across studies; in this context, it seems very relevant to standardize PFS measurement in definitive phase III trials. For example, in the Fidias trial, patients on the immediate docetaxel arm underwent radiologic assessment after cycles two, four and six, while patients in the delayed docetaxel arm the evaluation was performed every three months. Timing and the type of imaging studies used in the control arm has been considered one of the main limitations of this study, as unfavorably delaying detection of possible disease progression [37]. As it happens in routine daily practice, only about two thirds of patients on the control arm was able to receive second-line docetaxel, as opposed to 95% of patients who received the study drug in the immediate, maintenance arm; thus, the true benefit with "immediate" docetaxel in this study could be entirely attributed to the higher proportion of patients receiving active therapy in the maintenance setting. Indeed, a post-hoc analysis documented an identical OS duration of 12.5 months for patients who received docetaxel on either arm of the study, clearly indicating that when patients stop first-line chemotherapy, they should be followed closely to detect progression early and at a time when they remain fit for further treatment [24].

The benefit of maintenance therapy can be confounded by the absence of a predefined post-study treatment. Indeed, in JMEN trial (as well as in other ones) the discretion given to investigators in the choice of second-line therapy has been addressed as a major limitation, because it fails to provide any insight into the possibility that the benefit of maintenance therapy may be obtained also by the appropriate use of the same agent as salvage therapy at the time of disease progression. In that respect, the design of the Fidias' trial, with all patients receiving docetaxel as either maintenance or second-line treatment, appears to be a methodologically more correct study design to test the efficacy of a strategy introducing a non cross-resistant agent before progression. In the SATURN trial only a minority of patients assigned to placebo actually received an EGFR-TKI: with the current evidence, we do not know if the improvement in OS observed with maintenance erlotinib would have been the same, or reduced, if the study protocol had imposed cross-over after disease progression. Importantly, the adoption of a pre-specified, built-in second-line treatment option offers the advantage of reducing the proportion of patients who do not get access to further treatment, as demonstrated in the recently reported trial from Perol, in which more than 80% of patients in the observation arm received second-line pemetrexed [21,30,31].

Even if a bevacizumab maintenance in patients receiving bevacizumab combined with chemotherapy in the context of their first-line regimen is considered common practice on the basis of the registration trials, both of which maintained bevacizumab until progression after the completion of the assigned first-line regimen, with the notable exception of the recently-presented ovarian cancer trial clearly supporting the use of maintenance bevacizumab, this specific issue has never been assessed in *ad hoc *designed randomized trials [4,5,38]. Currently there are at least two trials designed to clarify its role in maintenance: the ECOG three-arm, phase III study of Paclitaxel/Carboplatin/Bevacizumab followed by randomization to pemetrexed versus bevacizumab versus pemetrexed/bevacizumab in non-squamous carcinoma and a study with Pemetrexed/Cisplatin/Bevacizumab followed by Pemetrexed/Bevacizumab versus Bevacizumab alone [39]. The approximately 4-month median PFS with single-agent erlotinib maintenance in the SATURN trial and 4.76 months with the combination of erlotinib and bevacizumab in the ATLAS trial, highlights the importance of establishing the relative contribution of each agent when a combination therapy strategy is being evaluated in the maintenance setting [31,32]. Another related question is whether subgroups of patients with specific clinico-pathological and/or molecular characteristics would especially benefit from the choice of a particular maintenance agent, among those currently available. Within the limits imposed by such methodological considerations, the only biomarker that clearly showed a statistically significant, quantitative interaction with the treatment assigned (erlotinib or placebo) was the presence of sensitizing EGFR mutations (p for interaction <.001); indeed, although even EGFR M- patients derive a small, but statistically significant, benefit in PFS from erlotinib maintenance (HR: 0.78, 95% CI: 0.63-0.96, p = 0.0185), the PFS gain of EGFR M+ patients is exceptionally wide (HR: 0.10, 95% CI: 0.04-0.25, p < 0.0001). The potential benefits of the inclusion of erlotinib in the maintenance treatment of EGFR M+ patients were consistent in the ATLAS trial, where erlotinib was combined with bevacizumab. However, at the moment there are no survival data and no further analyses of OS are planned, due to loss of patients to follow up [32]. In routine clinical practice obtaining information on EGFR mutational status is not always easy and time-consuming, being not exceptional that such information becomes available only when the patient is already receiving a standard first-line chemotherapy treatment: should this be the case, EGFR M+ patients have now the option to receive TKI right after the induction. The impact of erlotinib maintenance on OS of EGFR M+ patients, however, is currently uncertain. Survival data in EGFR M+ patients included in SATURN trial are not yet mature although the low number of EGFR M+ patients and the shape of the survival curves, make it unlikely that a statistically significant benefit will become apparent with longer follow up. It is true that EGFR TKI are effective in advanced NSCLC even when administered late in the course of the disease, but recent data document that about 50% of NSCLC patients treated with EGFR-TKIs will develop resistance-inducing EGFR mutations (such as the T790M) implying the possibility that resistant clones may expand as disease progresses [40-42]. Talking about costs in this specific context a recent retrospective cost-effectiveness analysis by Bradbury et al. reported the cost per year of life gained being not the most favorable in patients with sensitizing mutations in the EGFR gene. This was because these patients derived relatively greater benefit and stayed on treatment longer, thereby incurring considerably higher drug acquisition costs [43]. Besides EGFR mutations, histology represents a potentially crucial decision factor for the choice of specific maintenance agents. Currently, no direct comparisons between different agents in histology-selected subgroups of patients have been reported. In the JMEN trial, the benefit of maintenance pemetrexed is clearly confined to patients with non-squamous histology: indeed, in patients with squamous histology OS on pemetrexed maintenance was indistinguishable from that on placebo; conversely, in non-squamous patients pemetrexed maintenance resulted in a reduction of the risk of death of approximately 30% and prolonged median survival from 10.3 to 15.5 months [27]. In the SATURN trial, non-squamous patients on erlotinib maintenance experienced a 21% reduction in the risk of death and a prolongation of median survival from 10.5 to 13.7 months [31]. Similar results were obtained in the IFCT-GFPC trial (for which only PFS data are available), where the benefit for erlotinib maintenance was also confined to adenocarcinoma patients [21]. Conversely, in the ATLAS trial the benefit in OS gained from the addition of erlotinib to bevacizumab is very limited in both the adenocarcinoma and non-adenocarcinoma groups of patients (HR 0.91, 95% CI 0.74-1.12 and HR 0.98, 95% CI 0.64-1.49, respectively) [32]. Overall, in patients with non-squamous histology pemetrexed maintenance appears to provide the greatest benefit in terms of both PFS (HR 0.44) and OS (HR 0.70). Erlotinib also represents a reasonable choice (HR 0.60 and 0.79 for PFS and OS respectively) and may possibly be preferable in selected subgroups, such as females (HR 0.64 for erlotinib vs. HR 0.83 for pemetrexed) and east Asians patients (HR 0.66 for erlotinib vs. HR 1.05 for pemetrexed). An improvement in PFS was obtained with either erlotinib in patients with squamous histology in the SATURN trial (HR 0.76, 95% CI 0.60-0.95) or gemcitabine in patients with non-adenocarcinoma histology in the IFCT-GFPC trial (HR 0.56, 95% CI 0.37-0.85)[21,32]. Many other phase II and III trials are currently ongoing looking at maintenance therapy in NSCLC (Tables 3 and 4) [35,39,44,45]. Modulating the immune response in lung cancer is a strategy that is being actively investigated also in maintenance approach. The L-BLP25 (Stimuvax; Biomira Alberta, CA) is a liposome vaccine targeted to the extracellular core peptide of mucine 1 (MUC 1), a transmembrane protein expressed on epithelial cells. In a phase IIb trial, patients in stage III NSCLC, who had disease control after induction therapy, were randomized to receive vaccination weekly for 8 weeks and then they had the option to proceed to maintenance therapy, consisting in vaccination every 6 weeks or BSC. The median OS (primary endpoint) was 17.4 months for the vaccinated patients versus 13.0 months for those on BSC arm (p = 0.66)[46].

**Table 3:** New Phase II trials

Trial/Author	Comparison	Comments	References
**NCT00867009**	Pemetrexed and Cisplatin Plus Cetuximab Followed by Pemetrexed and Cetuximab as Maintenance IIIB or IV Nonquamous NSCLC	ongoing, but not recruiting	[39]

**NCT00687297**	Vandetanib (ZD6474)n With Docetaxel and Carboplatin Followed by Placebo or Vandetanib as Maintenance in IIIb, IV or Recurrent NSCLC	ongoing, but not recruiting	[39]

**NCT01004250**	Pemetrexed, Cisplatin, and Bevacizumab as Induction, Followed by Pemetrexed and Bevacizumab as Maintenance, in First-Line Nonsquamous Advanced NSCLC	currently recruiting	[39]

**NCT00425646**	Maintenance Strategy of Gleevce^® ^(Imatinib Mesylate) and Bevacizumab in Advanced, Non-Squamous, NSCLC Following Completion of First-Line Chemotherapy With Bevacizumab	ongoing, but not recruiting	[39]

**NCT00766246**	Docetaxel, Carboplatin and Bevacizumab as First-Line Treatment, Followed by Bevacizumab Plus Pemetrexed Versus Pemetrexed Alone as Second-Line Treatment of Stage IIIB or IV NSCLC	currently recruiting	[39]

**Table 4:** Current PHASE III trials

Trial/Author	Comparison	Comments	References
**NCT01107626 ECOG 5508**	Paclitaxel/Carboplatin/Bevacizumab, followed by pemetrexed vs bevacizumab vs pemetrexed/bevacizumab	not yet open for recruitment	[39]

**NCT00789373 Paz-Ares LG**	Maintenance Pemetrexed/BSC Vs BSC Immediately Following Induction Treatment With Pemetrexed + Cisplatin for Advanced Nonsquamous NSCLC	currently recruiting	[39]

**NCT00762034 Patel et al**.	Pemetrexed/Carboplatin/Bevacizumab Followed by Maintenance Pemetrexed/Bevacizumab vs Paclitaxel/Carboplatin/Bevacizumab Followed by Maintenance Bevacizumab in IIIB or IV Nonsquamous NSCLC	currently recruiting	[39]

**NCT00820755 NEXT**	Platinum-based chemotherapy plus cetuximab followed by cetuximab as maintenance with either 500 mg/m^2 ^every 2 w or 250 mg/m^2 ^every w	ongoing, not recruiting	[39]

**NCT00948675 Zinner et al**.	Pemetrexed/carboplatin with maintenance pemetrexed vs paclitaxel/carboplatin/bevacizumab with maintenance bevacizumab in IIIB or IV Nonsquamous NSCLC	currently recruiting	[39]

**NCT00693992 CALGB 30607**	Sunitinib as maintenance therapy vs placebo in Non-Progressing Patients Following 4 Cycles of Platinum-Based Combination in IIIB/IV NSCLC	currently recruiting	[39]

**NCT00961415 AVAPERL1**	Bevacizumab with or without pemetrexed as maintenance after 4 cycles Bevacizumab/Cisplatin/Pemetrexed	currently recruiting	[39]

**NCT00676507 STOP**	Lucanix™ (Belagenpumatucel-L) as Maintenance III/IV NSCLC with SD or PR and Who Have Responded to or Have Stable Disease Following One Regimen of Front-line, Platinum-based Combination Chemotherapy	currently recruiting	[39]

## Conclusions

Since no comparative trials of maintenance with different chemotherapy drugs or targeted agents have been conducted, no conclusive data are available yet of an advantage of maintenance therapy. As consequence, the choice and the duration of maintenance treatment remains largely empirical and needs to be explained and discussed with each patients in terms of current trials, different toxicity profiles (fatigue and myelosuppression on chemotherapy versus rash and diarrhea on EGFR TKis) or intra venous versus oral treatment options [47]. On the basis of the previous data in patients for whom maintenance therapy is deemed appropriate and who accepted to prolong treatment: i) switching to a non cross-resistant agent appears to provide greater benefit than continuing on one of the agents employed in the induction regimen (although critical information on this issue will be provided by the PARAMOUNT S124 trial, which investigated pemetrexed maintenance after pemetrexed/cisplatin induction and recently concluded enrollment) ii) in patients harboring sensitizing EGFR mutations, erlotinib (either alone or combined with bevacizumab for patients who have received bevacizumab as part of their primary treatment, if future data will state a benefit) currently appears to be the agent of choice iii) in patients with non-squamous/adenocarcinoma histology and with wt-EGFR or unknown mutational status, pemetrexed appears to provide the greatest advantage, although erlotinib, and to a lesser extent gefitinib (where available), may be reasonable alternatives for selected patients, taking into account the possible patient preference for an oral treatment option iv) patients with squamous histology and patients with KRAS mutations have limited treatment options and should be enrolled in specific clinical trials whenever possible.

## Abbreviations

Abbreviations are defined in the text where first used.

## Competing interests

The authors declare that they have no competing interests.

## Authors' contributions

All named authors conceived of the study, participated in its design and coordination and helped to draft the manuscript. All authors read and approved the final manuscript.

## References

[B1] Jemal A Siegel R Xu J Ward E Cancer statistics 2010 CA Cancer J Clin 2010 60 10.3322/caac.20073 20610543

[B2] Govindan R Page N Morgensztern D Read W Tierney R Vlahiotis A Spitznagel EL Piccirillo J Changing epidemiology of small cell lung cancer in the United States over the last 30 years: analysis of the surveillance, epidemiologic and end results database J Clin Oncol 2006 24 4539 4544 10.1200/JCO.2005.04.4859 17008692

[B3] Yang P Allen MS Aubry MC Wampfler JA Marks RS Edell ES Thibodeau S Adjei AA Jett J Deschamps C Clinical features of 5,628 primary lung cancer patients: experience at Mayo Clinic from 1997 to 2003 Chest 2005 128 452 462 10.1378/chest.128.1.452 16002972

[B4] Reck M Von Pawel J Zatloukal P Ramlau R Gorbounova V Leighl N J Mezger Archer V Moore N Manegold C Phase III trial of cisplatin plus gemcitabine with either placebo or bevacizumab as first-line therapy for non-squamous non-small cell lung cancer: AVAIL J Clin Oncol 2009 27 1227 1234 10.1200/JCO.2007.14.5466 19188680

[B5] Sandler A Gray R Perry MC Brhamer J Schiller JH Dowlati A Lilembaum R Johnson DH Paclitaxel-Carboplatin alone or with bevacizumab for non-small cell lung cancer New England J Med 2006 355 2542 2550 10.1056/NEJMoa061884 17167137

[B6] Pirker R Pereira Szczesna A jr Krzakowski M Ramlau R Vynnychenko I Park K Yu CT Ganul V Roh JK O'Byrne K de Marinis F Eberhardt W Goddemeier T Emig M Gatzemeier U Cetuximab plus chemotherapy in patients with advanced non-small-cell lung cancer (FLEX): an open-label randomized phase III trial Lancet 2009 373 1525 1531 10.1016/S0140-6736(09)60569-9 19410716

[B7] Sheperd FA Dancey J Ramlau R Mattson K Gralla R O'Rourke M Levitan N Gressot L Vincent M Burkes R Coughlin S Kim Y Berille J Prospective randomized trial of docetaxel versus best supportive care in patients with non-small-cell lung cancer previously treated with platinum-based chemotherapy J Clin Oncol 2000 18 2095 2103 10811675 10.1200/JCO.2000.18.10.2095

[B8] Fossella FV DeVore R Kerr RN Crawford J Natale RR Dunphy F Kalman L Miller V Lee JS Moore M Gandara D Karp D Vokes E Kris M Kim Y Gamza F Hammershaimb L Randomized phase III trial of docetaxel versus vinorlbine or ifosfamide in patients with advanced non-small cell lung cancer previously treated with platinum-containing chemotherapy regimens. The TAX 320 Non-Small-Cell Lung Cancer Study Group J Clin Oncol 2003 18 2354 2 10.1200/JCO.2000.18.12.2354 10856094

[B9] Hensing TA Schell MJ Lee JH Socinski MA Factors associated with the likelihood of receiving second line therapy for advanced non-small cell lung cancer Lung Cancer 2005 47 2 253 9 10.1016/j.lungcan.2004.07.040 15639724

[B10] Gridelli C Maione P Rossi A Ferrara ML Bareschino MA Schettino C Sacco PC Ciardiello F Potential treatment options after first line chemotherapy for advanced NSCLC: maintenance treatment or early second line? The Oncologist 2009 14 137 47 10.1634/theoncologist.2008-0152 19190239

[B11] NCCN practice guidelines in oncology v.2 2010 http://www.nccn.org

[B12] American Society of Clinical Oncology Clinical practice guidelines for the treatment of unresectable non-small cell lung cancer J Clin Oncol 1997 15 2996 3018 9256144 10.1200/JCO.1997.15.8.2996

[B13] Smith IE O'Brien ME Talbot DC Duration of chemotherapy in advanced non-small cell lung cancer: a randomized trial of three versus six courses of mitomycin, vinblastine and cisplatin J Clin Oncol 2001 19 1336 1343 11230476 10.1200/JCO.2001.19.5.1336

[B14] Socinski MA SChell MJ Peterman E Bakri K Yates S Gitten R Unger P Lee J Lee JH Tynan M Moore M Kies MS Phase III trial comparing a defined duration o therapy versus continuous therapy followed by a second-line therapy in advanced stage IIIB/IV non small cell lung cancer J Clin Oncol 2002 20 1335 1343 10.1200/JCO.20.5.1335 11870177

[B15] Von Plessen C Bergman B Andresen O Bremnes RM Sundstrom S Gilleryd M Stephens R Vilsvik J Aasebo U Sorenson S Palliative chemotherapy beyond three courses conveys no survival benefit or consistent quality of life benefits in advanced non small cell lung cancer Br J Cancer 2006 95 966 973 10.1038/sj.bjc.6603383 17047644 PMC2360695

[B16] Park JO Kim SW Ahn JS Suh C Lee JS Jang JS Cho EK Yang SH Choi JH Heo DS Yun YH Lee JW Park K Phase III trial of two versus four additional cycles in patients who are nonprogressive after two cycles of platinum-based chemotherapy in non-small cell lung cancer J Clin Oncol 2007 25 5233 5239 10.1200/JCO.2007.10.8134 18024869

[B17] Pfister DG Johnson DH Azzoli CG Sause W Smith TJ Baker SJr Olak J Stover D Strawn JR Turrisi AT Somerfield MR American Society of clinical Oncology treatment of unresectable non-small cell lung cancer guideline. Update 2003 J Clin Oncol 2004 22 330 353 14691125 10.1200/JCO.2004.09.053

[B18] Azzoli CG Baker S Termin S Pao W Aliff T Brahmer J Johnson DH Laskin JL Masters G Milton D Nordquist L Pfister DG Piantadosi S Schiller JH Smith R Smith YJ Strawn JR Trent D Giaccone G American Society of clinical Oncology practice guideline update on chemotherapy for stage IV Non-small cell lung cancer J Clin Oncol 2009 36 6251 6266 10.1200/JCO.2009.23.5622 PMC2793036 19917871

[B19] Brodowicz T Krzakowski M Zwitter M Tzekov V Ramlau R Ghilezn N Ciuleanu T Cucevic B Gyurkovits K Ulsperger E Jassem J Grgic M Saip P Szila M Oskina N Soldatenkova V Zielinsk C Wenczl M Cisplatin and gemcitabine first line chemotherapy followed by maintenance gemcitabine or best supportive care in advanced non-small cell lung cancer: a phase III trial Lung Cancer 2006 52 155 163 10.1016/j.lungcan.2006.01.006 16569462

[B20] Belani CP Waterhouse H Ghazal H Phase III study of maintenance gemcitabine (G) and best supportive care (BSC) versus BSC, following standard combination therapy with gemcitabine-carboplatin (G-Cb) for patients with advanced non-small cell lung cancer (NSCLC) J Clin Oncol 2010 28 15s abstr 7506

[B21] Perol M Chouaid C Milleron J Maintenance with either gemcitabine or erlotinib versus observation with predefined second-line treatment after cisplatin-gemcitabine induction chemotherapy in advanced NSCLC: IFCT-GFPC 0502 phase III study J Clin Oncol 2010 28 15s abstr 7507

[B22] Belani CP Barstis J Perry MC La Rocca RV Nattam SR Rinaldi D Clark R Mills GM Multicenter, randomized trial for stage IIIB or IV non small cell lung cancer using weekly paclitaxel or observation J Clin Oncol 2003 21 2933 2939 10.1200/JCO.2003.02.563 12885812

[B23] Goldie JH Coldman AJ A mathematic model for relating the drug sensitivity of tumours to their spontaneous mutation rate Cancer Treat Rep 1979 63 1727 17233 526911

[B24] Coate LE Shepherd FA Maintenance therapy in advanced non small cell lung cancer JTO 2010 5 5 723 734 10.1097/JTO.0b013e3181d86e8b 20354450

[B25] Westeel V Quoix E Moro Sibilot D Mercier M Breton JL Debieuvre D Richard P Haller MA Milleron B Herman D Level MC Puyraveau M Depierre A Randomized study of maintenance vinorelbine in responders with advanced non-small cell lung cancer J Natl Cancer Inst 2005 97 499 506 10.1093/jnci/dji096 15812075

[B26] Fidias PM Dakhil SR Lyss AP Loesch DM Waterhouse DM Bromund JL Chen R Kazmierski MH Treat J Obasaju CK Marciniak M Gill J Schiller JH Phase III study of immediate versus delayed docetaxel after front line therapy with gemcitabine plus carboplatin in advanced non-small cell lung cancer J Clin Oncol 2009 27 591 8 19075278 10.1200/JCO.2008.17.1405

[B27] Ciuleanu T Brodowicz T Zielinski C Kim JH Krzakowski M Laack E Wu YL Bover I Begbie S Tzekova V Cucevic B Pereira J Yang SH Madhavan J Sugarman KP Peterson P John WJ Krejcy K Belani CP Maintenance pemetrexed plus best supportive care vesus placebo plus best supportive care for non-small cell lung cancer: a randomized double-blind, phase III study Lancet 2009 374 1432 4 10.1016/S0140-6736(09)61497-5 19767093

[B28] Scagliotti GV Hanna N Fossella F Sugarman K Blatter J Peterson P Simms L Shepherd FA The differential efficacy of pemetrexed according to NSCLC histology, a review of two phase III studies Oncologist 2009 14 253 63 10.1634/theoncologist.2008-0232 19221167

[B29] Scagliotti GV Parikh P Von Pawel Biesma J Vansteenkiste J Manegold C Serwatowski P Gatzemeier U Digumarti R Zukin M Lee JS Mellemgaard A Park K Patil S Rolski J Goksel T de Marinis F Simms L PSugarman K Gandara D Phase III study comparing cisplatin plus gemcitabine with cisplatin plus pemetrexed in chemotherapy-naive patients with advanced non-small cell lung cancer J Clin Oncol 2008 26 3543 51 10.1200/JCO.2007.15.0375 18506025

[B30] Cappuzzo F Coudert BP Wierzbicki R Efficacy and safety of erlotinib as first-line maintenance in NSCLC following non-progression with chemotherapy: results from the phase III SATURN study Presented at the 13th World Conference on Lung Cancer, July 31 to August 4, 2009 abstract A2.1

[B31] Cappuzzo F Ciuleanu L Stelmakh L Cicenas S Szczésna A Juhász E Esteban E Molinier O Brugger W Melezínek I Klingelschmitt G Klughammer B Giaccone G Erlotinib as maintenance treatment in advanced non-small-cell lung ancer: a multicentre, randomized, placebo-controlled phase 3 study Lancet 2010 11 521 529 10.1016/S1470-2045(10)70112-1 20493771

[B32] Kabbinavar F Miller Va Johnson BE Overall survival in ATLAS, a phase IIIB study comparing bevacizumab therapy +/- Erlotinib after completion of chemotherapy with bevacizumab for first line treatment of locally advanced, recurrent metastatic non-small-cell lung cancer J Clin Oncol 2010 28 15s abstr 7526

[B33] Gaafar RM Surmont V Scagliotti GV A double-blind, randomized, placebo-controlled phase III intergroup study of gefitinib (G) in patients (pts) with advanced NSCLC, non-progressing after first-line platinum-based chemotherapy (EORTC 08021-ILCP 01/03) J Clin Oncol 2010 28 15s abstr 7518 10.1016/j.ejca.2011.06.045 21802939

[B34] Belani CP Dakhil S Waterhouse DM Clark RH Monberg MJ Ye Z Obasaju CK Randomized phase II trial of gemcitabine plus weekly versus three weekly paclitaxel in previously untreated advanced non small cell lung cancer Ann Oncol 2007 18 1 110 115 17043094 10.1093/annonc/mdl344

[B35] Paz-Ares LG Altug S Vaury AT Jaime JC Russo F Visseren-Grul C Treatment rationale and study design for a phase III, double-blind, placebo-controlled study of maintenance pemetrexed plus best supportive care versus best supportive care immediately following induction treatment with pemetrexed plus cisplatin for advanced nonsquamous non-small cell lung cancer BMC Cancer 2010 8 10 85 10.1186/1471-2407-10-85 PMC2847958 20211022

[B36] Klein R Wielage R Muehlenbein C Liepa AM Babineaux S Lawson A Schwartzberg L Cost-effectiveness of pemetrexed as first-line maintenance therapy for advanced non squamous non-small-cell-lung cancer J Thorac Oncol 2010 5 8 1263 72 10.1097/JTO.0b013e3181e15d16 20581708

[B37] Owokikonoko T Ramalingam SS Belani CP Maintenance therapy for advanced Non-small cell lung cancer: current status, controversies and emerging consensus Clin Cancer Res 2010 16 9 10.1158/1078-0432.CCR-09-2328 20388843

[B38] Burger MF Brady MA Bookman JL Phase III trial of bevacizumab (BEV) in the primary treatment of advanced epithelial ovarian cancer (EOC), primary peritoneal cancer (PPC), or fallopian tube cancer (FTC): A Gynecologic Oncology Group study J Clin Oncol (Meeting Abstracts) 2010 28 18 LBA1

[B39] Clinical Trials http://www.clinicaltrials.gov

[B40] Rosell R Moran T Queralt C Screening for Epidermal Growth Factor Receptor Mutations in Lung Cancer NEJM 2009 361 10 958 96 10.1056/NEJMoa0904554 19692684

[B41] Maheswaran S Sequist LV Nagrath S Ulkus L Brannigan B Collura CV Inserra E Iafrate AJ Bell DW Muzikansky A Irimia D Settleman J Tompkins RG Lynch TJ Toner M Haber DA Detection of Mutations in EGFR in Circulating Lung-Cancer Cells NEJM 2008 359 366 377 10.1056/NEJMoa0800668 18596266 PMC3551471

[B42] Rosell R Molina MA Costa C Outcome to erlotinib in non-small cell lung cancer (NSCLC) patients (p) according to the presence of the EGFR T790M mutation and BRCA1 mRNA expression levels in pretreatment biopsies J Clin Oncol 2010 28 15s abstr 7514

[B43] Bradbury PA Tu D Seymour L Impact of clinical and molecualr predictors of benefit from erlotinib in advanced non-small cell lung cancer on cot-effectiveness J Clin Oncol 2008 26 344s abstr 6531

[B44] Patel JD Bonomi P Socinski MA Govindan R Hong S Obasaju C Pennella EJ Girvan AC Guba SC Treatment Rationale and Study Design for the PointBreak Study: Randomized, Open-label Phase III Study of Pemetrexed/Carboplatin/Bevacizumab Followed by Maintenance Pemetrexed/Bevacizumab Versus Paclitaxel/Carboplatin/Bevacizumab Followed by Maintenance Bevacizumab in Patients with Stage IIIB or IV Nonsquamous Non-Small-Cell Lung Cancer Clinical Lung Cancer 2009 10 4 252 256 10.3816/CLC.2009.n.035 19632943

[B45] Zinner R Saxman S Peng G Randomized, open-label study of pemetrexed/carboplatin followed by maintenance pemetrexed versus paclitaxel/carboplatin/bevacizumab followed by maintenance bevacizumab in patients with advanced non-small cell lung cancer of nonsquamous histology J Clin Oncol 2010 28 15s TPS290 10.3816/CLC.2010.n.045 20837462

[B46] Butts C Murray N Maksymiuk A Goss G Marshall E Soulières D Cormier Y Ellis P Price A Sawhney R Davis M Mansi J Smith C Vergidis D Ellis P MacNeil M Palmer M Randomized phase IIb trial of BLP25 liposome vaccine in stage IIIB and IV non-small cell lung cancer J Clin Oncol 2005 23 6674 6681 10.1200/JCO.2005.13.011 16170175

[B47] Gandara DR Mack PC Lara PN Herbst RS Evolving treatment algorithms for advanced non-small-cell lung cancer:2009 Looking toward 2012 Clin Lung Cancer 2009 10 6 392 4 10.3816/CLC.2009.n.074 19900855

